# We need to chat about artificial intelligence

**DOI:** 10.5694/mja2.51992

**Published:** 2023-06-11

**Authors:** Enrico W Coiera, Karin Verspoor, David P Hansen

**Affiliations:** ^1^ Centre for Health Informatics Macquarie University Sydney NSW; ^2^ RMIT University Melbourne VIC; ^3^ Australian E‐Health Research Centre, CSIRO Brisbane QLD

**Keywords:** Artificial intelligence, Information services, eHealth

With the arrival of large language models such as ChatGPT, AI is reshaping how we work and interact

Long foretold and often dismissed, artificial intelligence (AI) is now reshaping how we work and interact as a society.^1^ For every claim that AI is overhyped and underperforming, only weeks or months seem to pass before a new breakthrough asks us to re‐evaluate what is possible. Most recent, it is the very public arrival of large language models (LLMs) such as the generative pre‐trained transformers (GPTs) in ChatGPT. In this perspective article, we explore the implications of this technology for health care and ask how ready the Australian health care system is to respond to the opportunities and risks that AI brings.

GPTs are a recent class of machine learning technology. Guided by humans who provide it with sample responses and feedback, ChatGPT was initially trained on 570 gigabytes of text, or about 385 million pages of Microsoft Word, and, at first release, the language model had 175 billion parameters.^2^ This massive model of the relationships between words is generative in that it produces new text, guided by the model, in response to prompts. It can answer questions, write songs, poems, essays, and software code. Other generative AIs such as DALL‐E, which is trained on images, can create startlingly good pictures, including fictitious or “deep fake” images of real people.^3^

Today's LLMs are story tellers, not truth tellers. They model how language is used to talk about the world, but at present they do not have models of the world itself. The sheer size of ChatGPT means that it can perform tasks it was not explicitly trained to do, such as translate between languages. ChatGPT amassed 100 million users in the first two months that it was available.^4^ So compelling are the linguistic skills of LLMs that some have come to believe such AI is sentient,^5^ despite the prevailing view that as statistical pattern generators, they cannot have consciousness or agency. Australian singer Nick Cave called ChatGPT “a grotesque mockery of what it is to be human” after seeing it generate new songs in his style.^6^

The health care uses of generative models will soon become clearer.^7^ Epic has agreed with Microsoft to incorporate its GPT‐4 model into their electronic health records, which have been used for over 305 million patients worldwide.^8^ LLMs are likely to find application in digital scribes, assisting clinicians to create health records by listening to conversations and creating summaries of the clinical content.^9^, ^10^ They can create conversational agents, which change the way we search medical records and the internet, synthesising answers to our questions rather than retrieving a list of documents.^11^

We should prepare for a deluge of articles evaluating LLMs on tasks once reserved for humans, either being surprised by how well the technology performs or showcasing obvious limits because of the lack of a deep model of the world.^12^ Especially when it comes to clinical applications, producing text or images that are convincing is not the same as producing material that is correct, safe, and grounded in scientific evidence. For example, conversational agents can produce incorrect or inappropriate information that could delay patients seeking care, trigger self‐harm, or recommend inappropriate management.^13^ Generative AI may answer patients’ questions even if not specifically designed to do so. Yet all such concerns about technology limitations are hostage to progress. It would be foolish indeed to see today's performance of AI as anything other than a marker on the way to ever more powerful AI.

## The unintended consequences of AI

It is the unintended consequences of these technologies that we are truly unprepared for. It was hard to imagine in the early innocent days of social media, which brought us the Arab Spring,^14^ just how quickly it would be weaponised. Algorithmic manipulation has turned social media into a tool for propagating false information, enough to swing the results of elections, create a global antivaccination movement, and fashion echo chambers that increasingly polarise society and mute real discourse.

Within two months of the release of ChatGPT, scientific journals were forced to issue policies on “non‐human authors” and whether AI can be used to help write articles.^15^ Universities and schools have banned its use in classrooms and educators scramble for new ways to assess students, including returning to pen and paper in exams.^16^ ChatGPT is apparently performing surprisingly well on questions found in medical exams.^17^

The major unintended consequences of generative models are still to be revealed.^18^ LLMs can produce compelling misinformation and will no doubt be used by malicious actors to further their aims. Public health strategies already must deal with online misinformation; for example, countering antivaccination messaging. Maliciously created surges of online messages during floods, heat events, and pandemics could trigger panic, swamp health services, and encourage behaviours that disrupt the mechanics of society.^19^

## The national imperative to respond to the challenges of AI

With AI's many opportunities and risks, one would think the national gaze would be firmly fixed on it. However, Australia lags most developed nations in its engagement with AI in health care and has done so for many years.^20^ The policy space is embryonic, with focus mostly on limited safety regulation of AI embedded in clinical devices and avoidance of general purpose technologies such as ChatGPT. Some clinical colleges and organisations have been more “fleet of foot”, adapting their training programs or developing frameworks for the ethical use of AI.^21^, ^22^ Yet there is currently no national framework for an AI‐ready workforce, overall regulation of safety, industry development, or targeted research investment. The national conversation on AI in health care has for now remained niche and low in priority.

Indeed, there has been a view in some quarters that all we need to do as a nation is adopt the best of what is produced internationally, and that we do not need deep sovereign capabilities. Nothing is further from the truth. Without some degree of algorithmic sovereignty (the capability to produce or modify AI in Australia), the nation is exposed to new risks and misses one of the most significant industrial revolutions of our times.^23^

We do not want to just export our clinical datasets and import back the models built with them. We should be a value adding economy that builds and exports these technologies ourselves. Australia's $1.4 billion clinical trials sector^24^ will face stiff international competition from those who use AI to identify, enrol and monitor patients more effectively and at a lower cost. Our health response to climate change will depend heavily on digital health and AI for mitigation and adaptation.^25^ Further, AI requires local customisation to support local practices and reflect diverse populations or health service differences.^9^ Without local capability, paying to modify clinical AI will likely become a huge burden on our health system. Critically, using AI requires retraining of the workforce, retooling health services, and transforming workflows. The health system is already resource‐constrained, and such changes will not happen without strategic investment.

The national discussion on what to do next has begun, with a roadmap for AI in health care produced by the Australian Alliance for AI in Healthcare (AAAiH) — a national collective of over 100 organisations including academia, industry, peak bodies, and health services providers.^26^ The roadmap is the product of feedback from 152 key stakeholder organisations and individuals and contains 24 recommendations across eight priority areas. The highest community priority identified was for health care AI to be safe for patients and developed and used ethically. AI privacy and data security were another major concern. Respondents identified the need for genuine whole‐of‐nation leadership in the health care AI space as well as robust governance of the sector. Gaps in our workforce capability to build and use health care AI were clearly identified, as was the need for consumers to be fully engaged in shaping the health care AI agenda. Respondents also rated the gaps in our capability to adopt AI into practice and the need to enhance local industry capability as issues needing clear attention. While a great start, the roadmap now needs to be converted into action, and that will require bringing together the skills and interests of many stakeholders, from government, consumer bodies, clinicians, industry, health service providers, and academia.

We can only expect the pace of AI innovation to accelerate, and for its consequences, good and ill, to multiply. We have a national imperative to both harness and benefit from these technologies, and not be hostages to the decisions of others. The time for urgent national engagement has arrived.

## Open access

Open access publishing facilitated by Macquarie University, as part of the Wiley – Macquarie University agreement via the Council of Australian University Librarians.

## Competing interests

Enrico Coiera is a shareholder and Board member of Evidentli, a digital health company.

## Provenance

Not commissioned; externally peer reviewed.

## References

[1] Coiera E. The fate of medicine in the time of AI. Lancet 2018; 392: 2331‐2332. 30318263 10.1016/S0140-6736(18)31925-1

[2] Tamkin A , Ganguli . How large language models will transform science, society, and AI [website]. Stanford University Human‐Centered Artificial Intelligence, 2021. https://hai.stanford.edu/news/how‐large‐language‐models‐will‐transform‐science‐society‐and‐ai (viewed May 2023).

[3] Taylor J. From Trump Nevermind babies to deep fakes: DALL‐E and the ethics of AI art. The Guardian 2022; 19 June. https://www.theguardian.com/technology/2022/jun/19/from‐trump‐nevermind‐babies‐to‐deep‐fakes‐dall‐e‐and‐the‐ethics‐of‐ai‐art (viewed May 2023).

[4] Milmo D. ChatGPT reaches 100 million users two months after launch. The Guardian 2023; 3 Feb. https://www.theguardian.com/technology/2023/feb/02/chatgpt‐100‐million‐users‐open‐ai‐fastest‐growing‐app (viewed May 2023).

[5] Tiku N. Google fired engineer who said its AI was sentient. The Washington Post 2022; 22 July. https://www.washingtonpost.com/technology/2022/07/22/google‐ai‐lamda‐blake‐lemoine‐fired/ (viewed May 2023).

[6] Cain S. “This song sucks”: Nick Cave responds to ChatGPT song written in style of Nick Cave. The Guardian 2023; 17 Jan. https://www.theguardian.com/music/2023/jan/17/this‐song‐sucks‐nick‐cave‐responds‐to‐chatgpt‐song‐written‐in‐style‐of‐nick‐cave (viewed May 2023).

[7] Lee P , Bubeck S , Petro J . Benefits, limits, and risks of GPT‐4 as an AI chatbot for medicine. N Engl J Med 2023; 388: 1233‐1239. 36988602 10.1056/NEJMsr2214184

[8] Edwards B. GPT‐4 will hunt for trends in medical records thanks to Microsoft and Epic. ars Technica 2023; 19 Apr. https://arstechnica.com/information‐technology/2023/04/gpt‐4‐will‐hunt‐for‐trends‐in‐medical‐records‐thanks‐to‐microsoft‐and‐epic/ (viewed May 2023).

[9] Coiera E , Liu S . Evidence synthesis, digital scribes, and translational challenges for artificial intelligence in healthcare. Cell Rep Med 2022; 3: 100860. 36513071 10.1016/j.xcrm.2022.100860 PMC9798027

[10] Navarro DF , Dras M , Berkovsky S ; editors. Few‐shot fine‐tuning SOTA summarization models for medical dialogues. Proceedings of the 2022 Conference of the North American Chapter of the Association for Computational Linguistics: Human Language Technologies: Student Research Workshop; Seattle, Washington (USA), and online; July 2022. ACL Anthology, 2022. https://aclanthology.org/2022.naacl‐srw.32/ (viewed May 2023).

[11] Laranjo L , Dunn AG , Tong HL , et al. Conversational agents in healthcare: a systematic review. J Am Med Inform Assoc 2018; 25: 1248‐1258. 30010941 10.1093/jamia/ocy072 PMC6118869

[12] Birhane A , Raji D . ChatGPT, Galactica, and the progress trap. WIRED 2022; 9 Dec. https://www.wired.com/story/large‐language‐models‐critique/ (viewed May 2023).

[13] Kocaballi AB , Quiroz JC , Rezazadegan D , et al. Responses of conversational agents to health and lifestyle prompts: investigation of appropriateness and presentation structures. J Med Internet Res 2020; 22: e15823. 32039810 10.2196/15823 PMC7055771

[14] Wolfsfeld G , Segev E , Sheafer T . Social media and the Arab Spring: politics comes first. International Journal of Press/Politics 2013;18: 115‐137.

[15] Flanagin A , Bibbins‐Domingo K , Berkwits M , Christiansen SL . Nonhuman “authors” and implications for the integrity of scientific publication and medical knowledge. JAMA 2023; 329: 637‐639. 36719674 10.1001/jama.2023.1344

[16] Cassidy C. Australian universities to return to “pen and paper” exams after students caught using AI to write essays. The Guardian 2023; 10 Jan. https://www.theguardian.com/australia‐news/2023/jan/10/universities‐to‐return‐to‐pen‐and‐paper‐exams‐after‐students‐caught‐using‐ai‐to‐write‐essays (viewed May 2023).

[17] Kung TH , Cheatham M , Medenilla A , et al. Performance of ChatGPT on USMLE: potential for AI‐assisted medical education using large language models. PLOS Digit Health 2023; 2: e0000198. 36812645 10.1371/journal.pdig.0000198 PMC9931230

[18] Harrer S. Attention is not all you need: the complicated case of ethically using large language models in healthcare and medicine. EBioMedicine 2023; 90: 104512. 36924620 10.1016/j.ebiom.2023.104512 PMC10025985

[19] Coiera E. The cognitive health system. Lancet 2020; 395: 463‐466. 31924402 10.1016/S0140-6736(19)32987-3

[20] Mackee N. AI in health care: Australia in danger of lagging behind. InSight+ 2020; 7 Sept. https://insightplus.mja.com.au/2020/35/ai‐in‐health‐care‐australia‐in‐danger‐of‐lagging‐behind/ (viewed May 2023).

[21] Royal Australian and New Zealand College of Radiologists . Ethical principles for AI in medicine. RANZCR, 2019. https://www.ranzcr.com/college/document‐library/ethical‐principles‐for‐ai‐in‐medicine (viewed May 2023).

[22] National Disability Insurance Scheme . Framework for artificial intelligence‐enabled assistive technology as supports under the NDIS [news release]. 7 Nov 2022. https://www.ndis.gov.au/news/8492‐framework‐artificial‐intelligence‐enabled‐assistive‐technology‐supports‐under‐ndis (viewed May 2023).

[23] Coiera E. Whoever controls the algorithms controls the world. The Mandarin 2020; 28 Sept. https://www.themandarin.com.au/140740‐opinion‐whoever‐controls‐the‐algorithms‐controls‐the‐world/ (viewed May 2023).

[24] MTPConnect . Australia's clinical trials sector. MTPConnect, 2021. https://www.mtpconnect.org.au/reports/clinicaltrialsreports2021 (viewed May 2023).

[25] Rahimi‐Ardabili H , Magrabi F , Coiera E . Digital health for climate change mitigation and response: a scoping review. J Am Med Inform Assoc 2022; 29: 2140‐2152. 35960171 10.1093/jamia/ocac134 PMC9667157

[26] Australian Alliance for AI in Healthcare . A Roadmap for AI in Healthcare for Australia [news release]. 1 Dec 2021. https://aihealthalliance.org/2021/12/01/a‐roadmap‐for‐ai‐in‐healthcare‐for‐australia/ (viewed May 2023).

